# The anti-depressive role of the Pei Yuan Kai Yu formula in cerebral small vessel disease based on gut microbiota

**DOI:** 10.3389/fphar.2025.1510250

**Published:** 2025-06-13

**Authors:** Lixuan Yang, Yutian Ao, Yannan Li, Baoan Dai, Junnan Li, Wenzhe Duan, Kaiqiang Dong, Zhenyun Han, Rongjuan Guo

**Affiliations:** ^1^ Department of Neurology, Beijing University of Chinese Medicine, Beijing, China; ^2^ Department of Neurology, Beijing Fengtai District Hospital of Traditional Chinese Medicine, Beijing, China; ^3^ Department of Neurology, Johns Hopkins University of Medicine, Baltimore, MD, United States; ^4^ Department of Neurology, Shenzhen Hospital of Beijing University of Chinese Medicine, Shenzhen, Guangdong, China; ^5^ Department of Neurology, Dongfang Hospital Beijing University of Chinese Medicine, Beijing, China

**Keywords:** Pei Yuan Kai Yu formula, cerebral small vessel disease, depression, microglia, inflammation, gut microbiota

## Abstract

**Background:**

Cerebral small vessel disease (CSVD), characterized by pathological changes in brain vessels, is a common cause of death in the elderly and often accompanied by depression, which significantly affects patients’ quality of life and rehabilitation; understanding its pathogenesis and developing innovative therapies are urgently needed, especially considering the role of the blood - brain barrier impairment and the gut - microbiota - gut - brain axis in this complex condition.

**Methods:**

Dahl/SS rats were fed a diet containing 8% NaCl and were subjected to chronic unpredictable mild stress (CUMS) stimulation for 4 weeks. PY (1.407 g/kg/day) was administered intragastrically to evaluate its role in CSVD with depression. Pseudo germ-free rats were colonized with gut microbiota from high-salt-fed rats exposed to CUMS, followed by PY administration.

**Results:**

In rats with CSVD and depression, PY significantly increased body weight; alleviated depression-like behaviors; and decreased the levels of inflammatory cytokines such as TNF-α, IL-1β, and IL-6 in both serum and hippocampus. Additionally, PY reversed inflammation-induced nerve damage; reduced the overexpression of microglia in the hippocampus; decreased the levels of hippocampal VEGF and MMP-9, and increased the levels of hippocampal occludin, ZO-1, and claudin-5. Moreover, PY improved the composition of gut microbiota and enhanced microbial diversity. PY induced characteristic changes in the microbiome, which were associated with inflammation, endothelial dysfunction, and depressive-like behaviors. These significant metabolites were identified and were found closely related to inflammation, endothelial cell dysfunction, and depression-like behaviors.

**Conclusion:**

In conclusion, PY acts as an antidepressant to slow down the progression of CSVD by inhibiting microglial activation, reducing inflammation and ameliorating endothelial dysfunction. It exerts its effect, at least in part, by enhancing microbiota-mediated metabolism *in vivo*.

## 1 Introduction

Cerebral small vessel disease (CSVD) is defined as pathological changes in arterioles, perforating arteries, capillaries, and venules in the brain (Mustapha et al., 2019). The clinical symptoms and radiological images of CSVD are heterogeneous. CSVD is a common cause of death in the elderly. A longitudinal study on aging reported that the 5-year mortality of CSVD was 8.4% (Lee et al., 2021). Among the CSVD patient population, depression, as a common complication, significantly affects the patients’ quality of life and the rehabilitation processes. For instance, a meta-analysis indicated that the destruction of white matter hyperintensities induced by CSVD increased the risk of depression (Fang et al., 2020). A questionnaire survey study reported that the depression increased the risk of disability and mortality in patients (Xia W. et al., 2022). However, heterogeneous pathogenesis weakens the efficacy of known therapeutic methods. Thus, a comprehensive understanding of the pathogenesis of CSVD complicated by depression is required, and innovative therapeutic approaches need to be developed.

Impairment of the blood-brain barrier (BBB) stands as a crucial pathological hallmark of CSVD (Xia Y. et al., 2022). The integrity of the BBB hinges on the intricate interplay among endothelial cells, microglial cells and other components (Kaplan et al., 2020). Therefore, the damage inflicted on the BBB by microglia encompasses these different components. Once the disruption of the BBB is triggered by early-stage endothelial inflammation, the body’s internal inflammatory balance is disturbed, and immune cells are activated to release a series of substances (Li et al., 2024). Cytokines, chemokines, and matrix metalloproteinases then trigger inflammatory cascades, thereby undermining the integrity of the BBB (Low et al., 2019). Once inflammatory mediators penetrate into the brain tissue, they prompt the activation of microglia (Gao and Hernandes, 2021). Subsequently, activated microglia discharge pro-inflammatory mediators, further aggravating the impairment of BBB, neuroinflammation, and depression (Ronaldson and Davis, 2020; Fang et al., 2023). Notably, the complex interplay does not stop at the BBB-related inflammation. The microbiota-gut-brain axis also plays a crucial role in the context of CSVD accompanied by depression.

Numerous prior studies have documented the relationship among the microbiota-gut-brain axis, neuroinflammation, and depression. Abnormalities in the gut microbiota can affect the pathogenesis of CSVD accompanied by depression (Reyes-Martínez et al., 2023; Saji et al., 2021; Simpson et al., 2021). Microbial-derived metabolites play a key role in the metabolic and signaling mechanisms of BBB, contributing to the defense against pathological conditions and the associated inflammation (Shi et al., 2023). However, this disruption of the intestinal ecosystem can undermine the overall immune system and the production of neuroprotective metabolites, thus exacerbating neuroinflammation and dysfunction of the BBB (Shi et al., 2023). Additionally, the systemic inflammation triggered by gut dysbiosis can promote the accumulation of pro-inflammatory cytokines in the central nervous system. Subsequently, it can damage microglia, thus triggering symptoms of depression (Reyes-Martínez et al., 2023). Therefore, targeting gut microbiota-mediated inflammation holds promise as a therapeutic approach for CSVD accompanied depression.

Traditional Chinese medicine (TCM) is an important part of complementary and alternative medicine, attracting the attention of researchers worldwide. Some studies have explored the potential of TCM in preventing and treating CSVD (Sun et al., 2022; Wang et al., 2023). TCM formulas usually consist of multiple medicinal substances. These substances act on different organs via multiple targets, exert therapeutic effects, and have relatively low side effects (Sun et al., 2022; Zhao et al., 2024). The Pei Yuan Kai Yu formula (PY) is a newly developed TCM formula. The main medicinal ingredients of PY are *Panax ginseng*, *Morinda officinalis*, *Hypericum perforatum*, *Cyperus rotundus*, *Cnidium officinale*, *Atractylodes lancea*, *Gardenia jasminoides*, and medicated leaven. Some of these ingredients have therapeutic effects on inflammation, vascular damage, and depression. For example, Li et al. found that the natural extract of *M. officinalis* could inhibit the NLRP3 inflammasome in stroke-stricken rats, thus alleviating the microglia-mediated depressive-like symptoms (Li Z. et al., 2021; Li et al., 2014). Besides, Farahani and Hashemi found the protective role of *C. rotundus* L. extract in cerebral vessels (Farahani and Hashemi, 2016). They discovered that *C. rotundus* L. extract can upregulate the expression of Bcl-x1, an anti-apoptotic gene, thus inhibiting inflammation-related damage and vascular impairment. Ginsenoside Rc, an extract of *P. ginseng*, maintains vascular homeostasis by regulating the expression of the angiotensin II pathway in endothelial cells (Wang et al., 2021). Additionally, some medicinal components of PY can target the gut microbiota to improve the pathological changes during disease progression. For example, *Panax notoginseng* and ginsenosides (active ingredients of *P. ginseng*) can regulate the composition and function of the gut microbiota, inducing the formation of a microbial phenotype that contributes to the repair of the intestinal barrier and cell reconstruction (Zhou et al., 2021; Xu et al., 2020; Huang et al., 2022). Some traditional Chinese medicines can promote the enrichment of different gut microbiota, thereby improving depressive-like behaviors and pathological changes in the brain (Qu et al., 2019; Han et al., 2021; Li et al., 2022a; Li et al., 2023). Thus, PY can regulate the gut microbiota of individuals suffering from CSVD with depression.

In this study, we put forward a novel hypothesis that, based on the gut microbiota, PY can regulate the metabolites associated with depression, thus alleviating depressive symptoms in patients with CSVD. To mimic CSVD with depression, Dahl/Salt-sensitive rats were selected and exposed to a high salt diet and chronic significant mental stress for 4 weeks. During this time, 1.407 g/kg/day of PY was administered intragastrically for 4 weeks as well. We recorded the changes in microglia, neurons, and endothelial cells using histopathological examinations and molecular biology techniques. Additionally, the mechanism of action of PY was verified through the fecal microbiota transplantation model.

## 2 Results

### 2.1 PY improved depressive-like behaviors

To simulate CSVD accompanied by depression, we induced depressive-like behaviors in Dahl/SS rats. The rats were fed a diet containing 8% NaCl and subjected to chronic unpredictable mild stress (CUMS) for consecutive weeks. During this 4-week CUMS induction period, the rats were intragastrically administered PY at a dose of 1.407 g/kg/day. Escitalopram (ESC) was employed as a positive control for PY.

**FIGURE 1:**
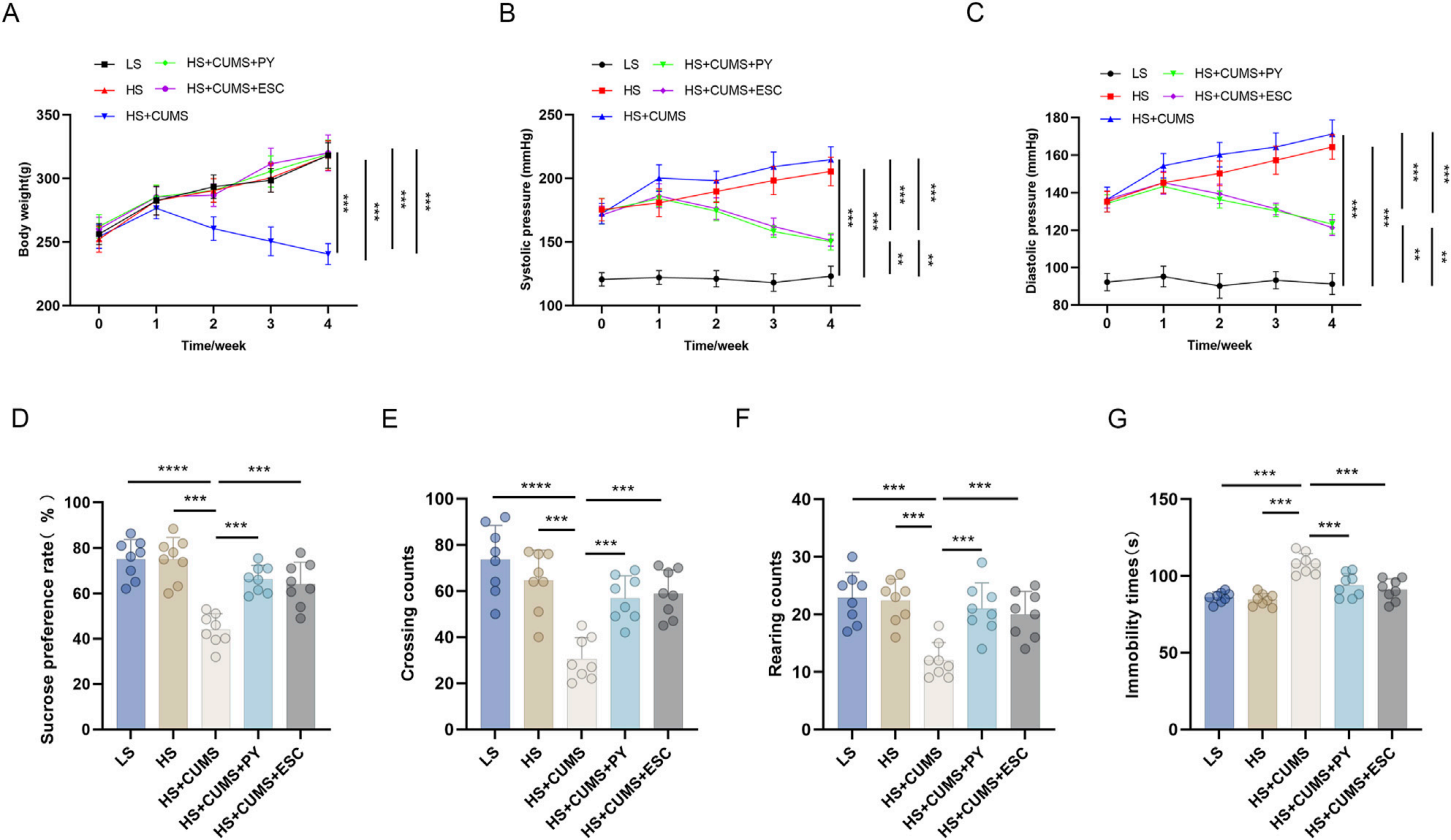
PY improved depressive-like behavior in rats and inhibited the elevation in blood pressure induced by a high-salt diet in combination with CUMS (*n* = 8). To simulate cerebral microvascular disease, rats in the high-salt group were fed a diet containing 8% NaCl for 4 weeks. While rats in the low-salt group were fed a diet containing 0.3% NaCl. Additionally, rats on a high-salt diet were subjected to chronic unpredictable mild stress (CUMS) for 4 weeks. During the 4 weeks of CUMS induction period, the rats were administered with 1.407 g/kg/day of PY or 1.05 g/kg/day of ESC via intragastric gavage. ESC was used as the positive control of PY. The indicators in the figure are as follows: **(A)** Body weight; **(B)** Systolic pressure; **(C)** diastolic pressure; **(D)** Sugar preference; **(E)** Crossing count in the open-field test; **(F)** Rearing count in the open-field test; **(G)** Immobility time in the forced-swim test. **P < 0.01, ***P < 0.001, and ****P < 0.0001.

Rats receiving only high-salt treatment exhibited no change in body weight (Figure 1A), however, their systolic and diastolic blood pressures were significantly higher compared to those on a low-salt diet (Figures 1B,C). These high-salt-fed rats showed no alterations in sugar preference (Figure 1D), the number of crossings and rearing counts in the open-field test (OFT) (Figures 1E,F), and the immobility time (Figure 1G) in the forced-swim test (FST).

CUMS led to a decrease in the body weight (Figure 1A) and an increase in blood pressure (Figures 1B,C) of the high-salt-fed rats. Moreover, CUMS triggered depressive-like behaviors in these rats, as manifested by a reduced sugar preference (Figure 1D), fewer crossing and rearing counts (Figures 1E,F), and an extended immobility time (Figure 1G). These changes were comparable to those observed after ESC treatment.

Treatment with PY increased the body weight of the rats that received both high-salt treatment and CUMS (Figure 1A), yet inhibited the blood pressure changes induced by high-salt and CUMS (Figures 1B,C). Additionally, PY increased sugar preference (Figure 1D), the number of crossings (Figure 1E), and rearing counts (Figure 1F), and simultaneously decreased the immobility time (Figure 1G) of the rats under CUMS and on a high-salt diet.

Collectively, these results demonstrated that PY can alleviate depressive symptoms, without exerting any effect on hypertension symptoms.

### 2.2 PY alleviated the over-activation of microglia and protected the BBB

Firstly, we compared the cell-related changes among different groups of rats. In the LS group as the control, the tissue structure showed normal characteristics. However, in the high-salt group, there was a clear increase in IBA-1 labeled microglia and GFAP, decrease in NeuN-labeled neurons, Nissl corpuscles, and myelin sheath, and damaged vascular endothelium.

**FIGURE 2:**
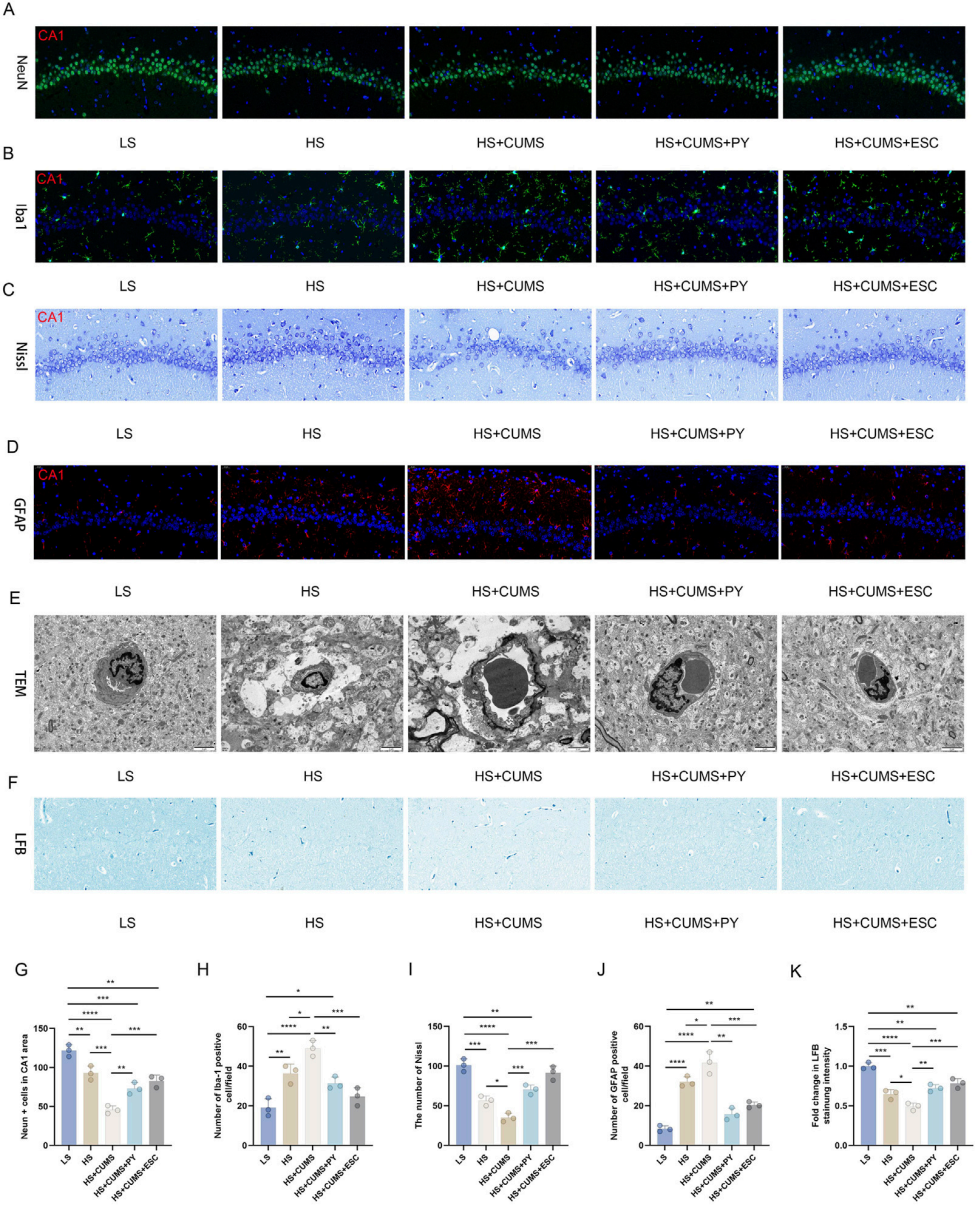
PY reversed the damage to neurons in the CSVD accompanied with depression. Hippocampal tissues were collected from rats in the following groups: low-salt, high-salt, high-salt + CUMS, high-salt + CUMS + PY, and high-salt + CUMS + ESC. Subsequently, immunostaining, histological staining, and transmission electron microscopy were carried out. **(A)** NeuN-labeled neurons; **(B)** IBA-1-labeled microglia; **(C)** Nissl staining for visualizing Nissl corpuscle; **(D)** GFAP-labeled astrocytes; **(E)** Transmission electron microscopy for evaluating the vascular endothelium; **(F)** Luxol Fast Blue (LFB) staining for myelin sheath; **(G)** NeuN + cells in the CA1 area; **(H)** Number of IBA-1 positive cells; **(I)** The number of Nissl bodies; **(J)** The number of GFAP positive cells; **(H)** Fold change in LFB staining intensity. For quantitive data presented in **(G–K)** the *n* = 8 was applied. *P < 0.05, **P < 0.01, ***P < 0.001, and ****P < 0.0001.

CUMS significantly exacerbated the inflammation and nerve injury. This was evident from multiple aspects: a notable decrease in the number of neurons, a substantial increase in the proportion of activated microglia, a visible reduction in the quantity of Nissl corpuscles, a marked decline in the integrity of myelin sheath, and severe destruction of vascular endothelium. In contrast, treatment with PY and ESC effectively mitigated the over-activation of microglia and safeguarded the BBB in rats that were fed a high-salt diet and subjected to CUMS (Figure 2).

**FIGURE 3:**
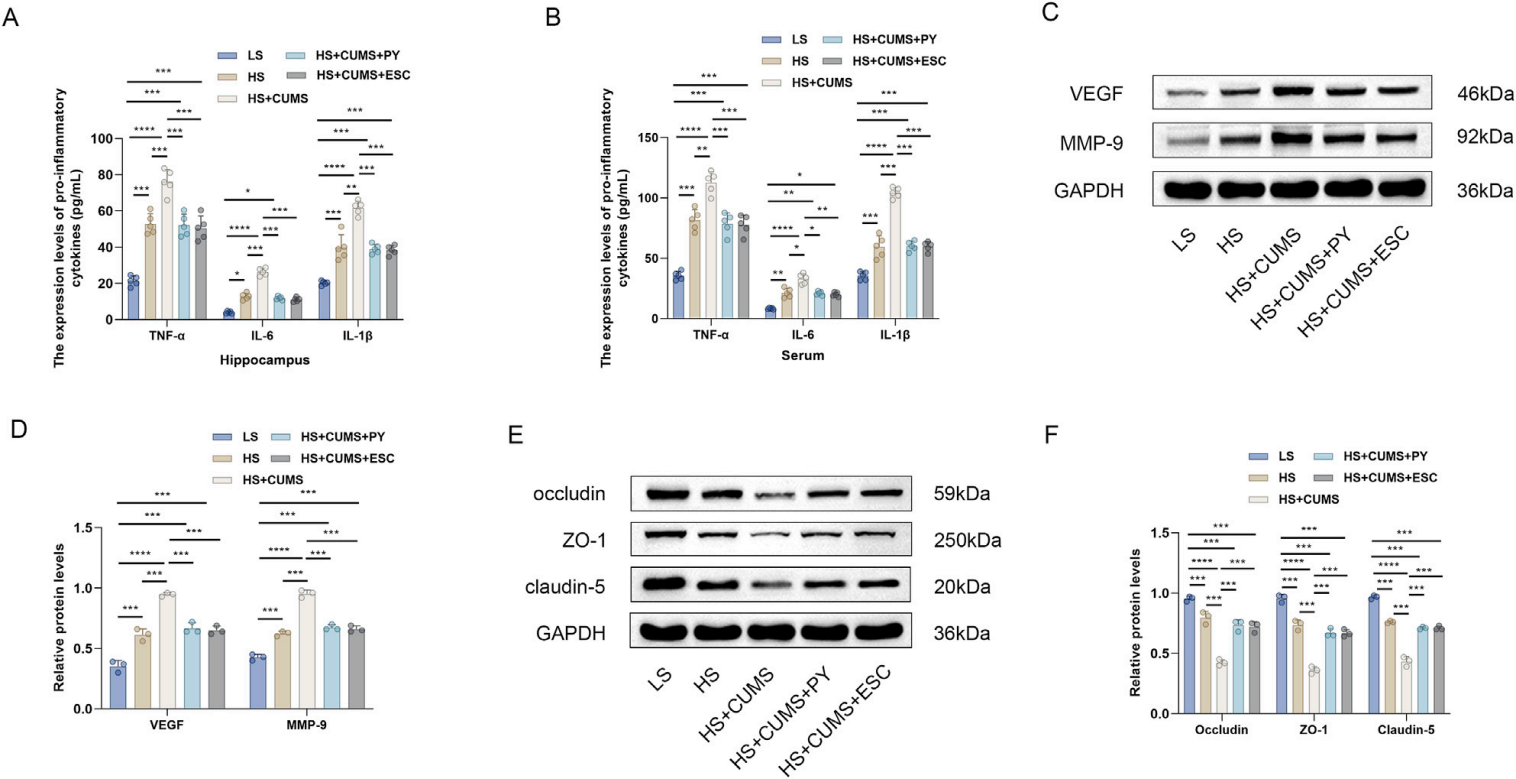
PY ameliorated endothelial dysfunction in the context of CSVD complicated by depression. Hippocampus and serum samples were collected and subjected to enzyme-linked immunosorbent assay (ELISA, *n* = 5) and Western blotting analysis (*n* = 3). **(A)** The expression levels of pro-inflammatory cytokines in the hippocampus were determined by ELISA; **(B)** The levels of pro-inflammatory cytokines in the serum were determined by ELISA; **(C)** The expression levels of VEGF and MMP-9 in the hippocampus were analyzed by Western blotting; **(D)** Protein levels of VEGF and MMP-9; **(E)** The expression levels of occludin, ZO-1, and claudin-5 in the hippocampus were detected by Western blotting; **(F)** Protein levels of occludin, ZO-1, and claudin-5. *P < 0.05, **P < 0.01, ***P < 0.001, and ****P < 0.0001.

Next, we measured the expression of pro-inflammatory cytokines in the hippocampus and serum, including TNF-α, IL-1β, and IL-6. High-salt treatment significantly increased the levels of these pro-inflammatory cytokines in the hippocampus and serum (Figures 3A,B). Rats fed a high-salt diet and subjected to CUMS, showed higher concentrations of pro-inflammatory cytokines compared with those fed with high-salt diet only (Figures 3A,B). Treatment with PY and ESC inhibited the increase in the levels of TNF-α, IL-1β, and IL-6 (Figures 3A,B), suggesting that PY and ESC played an anti-inflammatory role in the blood and nervous system.

We also measured the expression levels of endothelial function related factors, including vascular endothelial growth factor (VEGF) matrix metalloproteinase-9 (MMP-9), occludin, zonula occludens-1 (ZO-1), and claudin-5, to evaluate the vascular protection ability of PY (Figures 3C-F). High-salt treatment upregulated the levels of VEGF and MMP-9 and downregulated the levels of tight junction-related proteins (occludin, ZO-1 and claudin-5). CUMS aggravated the abnormal expression levels of VEGF, MMP-9, occludin, ZO-1, and claudin-5 in rats fed a high-salt diet. However, the abnormalities in the levels of these proteins disappeared after treatment with PY and ESC.

### 2.3 PY influenced gut microbiota during disease progression

#### 2.3.1 Pseudo-germ-free rat model construction and verification

Before investigating how PY influenced gut microbiota during disease progression, we successfully constructed a pseudo-germ-free rat model. As shown in Supplementary Figure S1, the number of colonies in the “Pseudo-germ-free rat” group was remarkably lower than that in the control group, validating the successful clearance of flora in the internal environment of these rats. This successful model construction provides a reliable foundation for the subsequent exploration of whether the gut microbiota mediated the effect of PY in the development of CSVD accompanied by depression.

#### 2.3.2 Impact of PY on gut microbiota in disease progression

**FIGURE 4:**
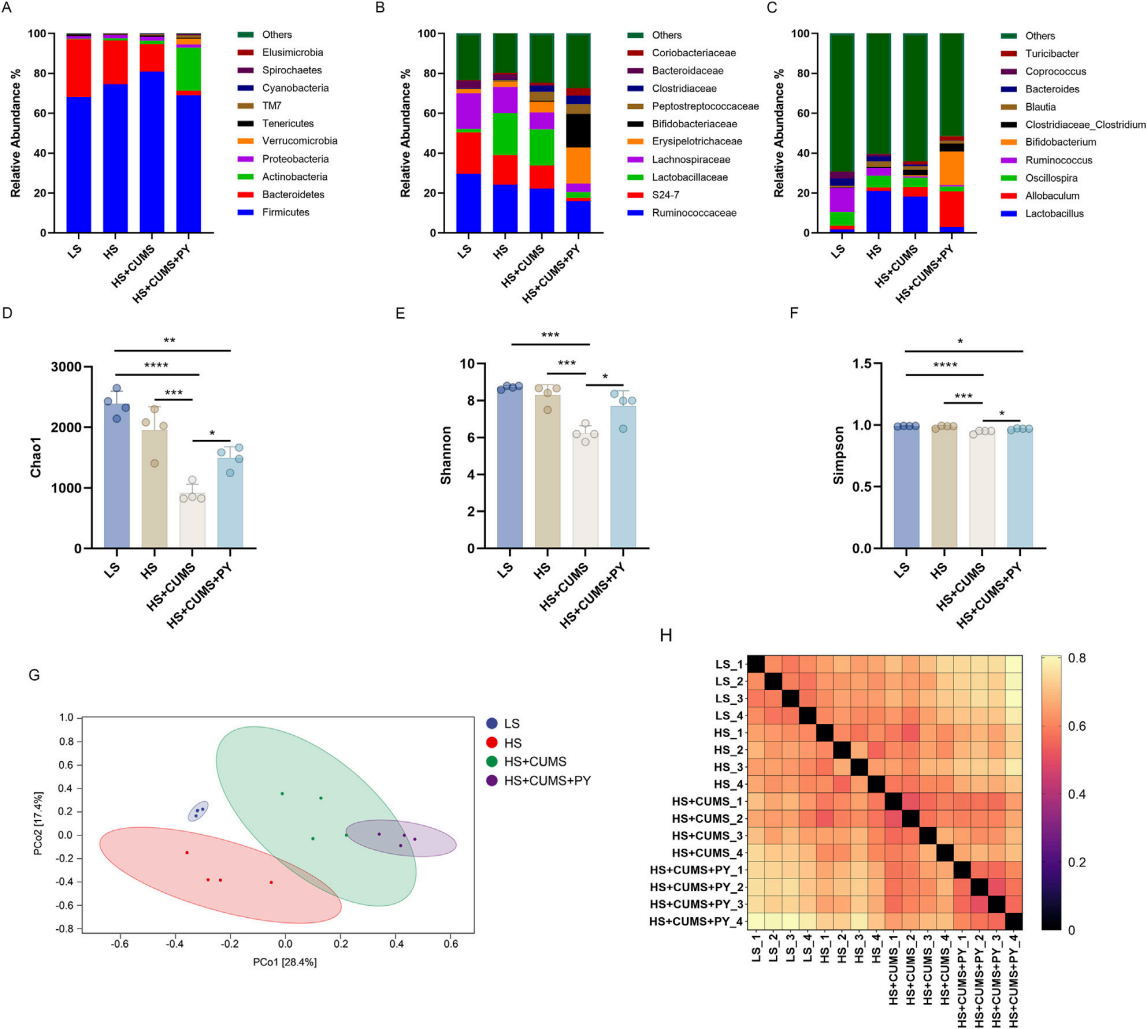
PY modified the composition of the gut microbiota during the progression of CSVD accompanied by depression. To elucidate the role of PY in modulating the gut microbiota in the context of CSVD with depression, fresh fecal samples were collected from the rats for 16S rDNA sequencing and subsequent microbiome analysis. **(A)** The composition of gut microbiota at the phylum level; **(B)** The composition of gut microbiota at the genus level; **(C)** The composition of gut microbiota at the genus level; **(D)** Chao1 α-diversity index, which reflects the richness of the gut microbiota, *n* = 4; **(E)** Shannon diversity index, considering both the richness and evenness of the gut microbiota, *n* = 4; **(F)** The Simpson diversity index, measuring the dominance and evenness of the gut microbiota, *n* = 4; **(G)** PCoA for β-diversity analysis based on Weighted UniFrac, visualizing the differences in the composition of the gut microbiota among samples; **(H)** The sample distance calculated based on the Weighted UniFrac, indicating the dissimilarity of the gut microbiota between different samples. *P < 0.05, **P < 0.01, ***P < 0.001, and ****P < 0.0001.

We investigated whether the gut microbiota mediated the effect of PY in the development of CSVD accompanied by depression. Fresh fecal samples were collected from rats for 16S rDNA sequencing. Amplicon sequence variants (ASVs) were obtained using the DADA2 plugin, in Quantitative Insights Into Microbial Ecology (QIIME2). The gut microbiota was annotated by aligning the ASVs with the reference sequences in the Greengenes database. Firmicutes and Bacteroidota were the top two phyla in the gut microbiota. At the family and genus levels, significant changes were observed in the composition of the gut microbiota (Figures 4A–C). The α-diversity metrics, including Chao1, Shannon, and Simpson indices, were calculated to characterize the alterations in richness, diversity, and evenness. Compared with the low-salt diet group, the high-salt diet group showed no changes in the α-diversity metrics (Figures 4D–F). However, CUMS decreased the Chao1, Shannon, and Simpson indices in the rats fed a high-salt diet, indicating that the α-diversity decreased during the progression of accompanied by depression (Figures 4D–F). To analyze the β-diversity, we calculated the dissimilarity distance based on the weighted UniFrac method (Figure 4H). We used the weighted UniFrac distance in a principal coordinate analysis (PCoA) dimensionality-reduction method to visualize the distances between experimental groups (Figure 4G). The HS + CUMS + PY group and the HS + CUMS group, though partially overlapping, had non-coincident distribution centers. This, along with the significant differences among the four groups, indicates that both the disease model and the intervention of PY had an impact on the β-diversity of the gut microbiota.

**FIGURE 5:**
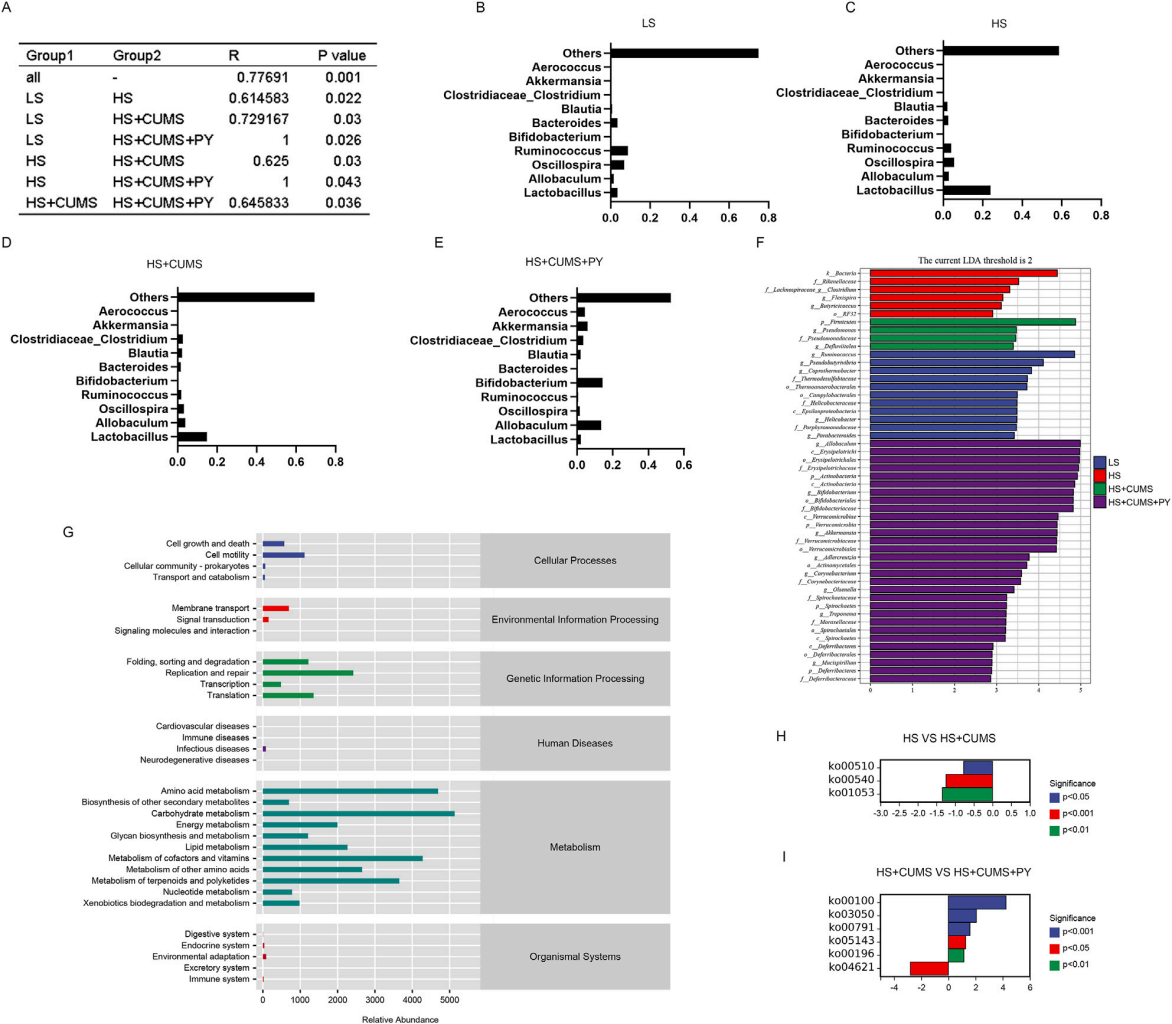
Screening, identification, and functional prediction of significant microbiota. **(A)** Depiction of the community difference among the samples (*n* = 8); **(B–E)** Presentation of the top 10 most abundant microbes in each respective group; **(F)** Application of linear discriminant analysis effect size (LEfSe) for the identification of marked microbiota, which helps to pinpoint the characteristic microbial taxa; **(G)** Visualization of the relative abundance of metabolic pathways based on PICRUSt 2, offering a view on the functional potential of the microbiota; **(H,I)** Illustration of the significant metabolic pathways, highlighting the key metabolic processes associated with the microbiota under study.

We also determined the differences in the microbial communities among the four groups through analysis (Figure 5A). Based on microbial abundance, the top 10 microbes in the low-salt (LS) group, high-salt (HS) group, high-salt + CUMS (HS + CUMS) group, and high-salt + CUMS + PY (HS + CUMS + PY) group were identified subsequently (Figures 5B–E). Then we used linear discriminant analysis effect size (LEfSe) to determine the characteristic microbes in the four groups (Figure 5F). At the genus level, we identified bacteria such as *Allobaculum*, *Bifidobacterium*, *Akkermansia*, *Adlerceutzia*, *Corynebacterium*, *Treponema*, and *Mucispirillum,* as the microbial markers in the PY group. In the HS + CUMS group, bacteria *Pseudomonas* and *Defluviitalea* were significantly enriched. CUMS modulated the enrichment of the microbiota, promoting depressive symptoms in high-salt-fed rats, while the drug intervention (PY) improved the pathological condition by targeting the abnormally distributed microbiota. Therefore, these microorganisms played a key role in identifying the therapeutic effects of PY on CSVD. We used the PICRUSt two tool to determine the functions mediated by the microbiota based on the Kyoto Encyclopedia of Genes and Genomes (KEGG) database. For example, we found that the microbial markers were associated with cell motility, membrane transport, DNA replication and repair, amino acid metabolism, carbohydrate metabolism, metabolism of cofactors and vitamins, and metabolism of terpenoids and polyketides (Figure 5G). Compared with their activities in the HS group, the KEGG pathways, including ko00510, ko00540, and ko01053, were downregulated in the HS + CUMS group. Compared with the activities in the HS + CUMS group, the ko00100, ko03050, ko00791, ko05143, and ko00196 pathways were upregulated, while the ko04621 pathway was downregulated in the HS + CUMS + PY group (Figures 5H,I).

We determined the relationship between gut microbiota and environmental factors. Through Spearman correlation analysis, the correlations between the gut microbiota in the HS + CUMS and the HS + CUMS + PY groups and environmental factors, including pro-inflammatory cytokines, tight junction-related proteins, VEGF, MMP-9, and depressive-like behaviors were evaluated.

**FIGURE 6:**
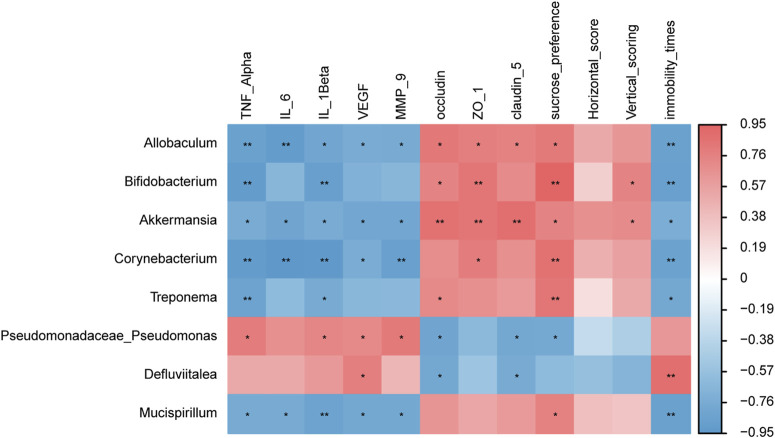
The correlation between signature microbiota and environmental factors (*n* = 8). Spearman correlation analysis was performed to determine the relationship between signature microbiota and inflammation, endothelial dysfunction, and depressive-like behaviors. *: Statistically significant at P ≤ 0.05. **: Statistically significant at P ≤ 0.01.

In the HS + CUMS + PY group, a certain type of bacterium *Akkermansia* was positively correlated with occludin, ZO-1, claudin 5, sucrose preference, and vertical scoring, but negatively correlated with TNF-α, IL-6, IL-1β, VEGF, MMP-9, and immobility time (Figure 6). *Allobaculum* was negatively correlated with TNF-α, IL-6, IL-1β, VEGF, MMP-9, and immobility time, but positively correlated with occludin, ZO-1, claudin 5, and sucrose preference (Figure 6). In the HS + CUMS group, *Pseudomonas* was significantly correlated with TNF-α, IL-1β, VEGF, MMP-9, occludin, claudin 5, and sucrose preference (Figure 6).

#### 2.3.3 Supplementary experiment on the impact of PY and gut microbiota on rat physiology and behavior

Before delving into the effects of PY and gut microbiota on rat physiology and behavior, it is crucial to first introduce the pseudo-germ-free rat model used in this experiment.

For the physiological parameters, as shown in Supplementary Figures S2A–C, compared with the HS + CUMS group, the pseudo-germ-free rat + HS-CUMS + PY group exhibited a certain improving effect on the body weight and blood pressure of rats, but the improving effect was not significant compared to the HS + CUMS group. Regarding behavioral parameters (Supplementary Figures S2D–G), the data revealed that while PY showed some capacity to improve the behavioral indices in pseudo-germfree rats, this improvement was not statistically significant. By contrast, PY remarkably improved the behavior of rats that only received HS + CUMS treatment. All these results imply that the effectiveness of PY in modifying body weight, blood pressure and behavioral patterns may be closely associated with the gut microbiota. It is likely that PY exerts its full-fledged effect on rat behavior through modulating the gut microbiota, rather than acting independently.

### 2.4 PY modulated microbiota-mediated metabolic pathways

The gut microbiota has the ability to generate either beneficial or harmful metabolites, which in turn modulate the metabolic activities within the body. To evaluate whether PY can ameliorate depressive-like behaviors and endothelial dysfunction, the levels of microbiota-related metabolites were measured in the plasma of rats.

**FIGURE 7:**
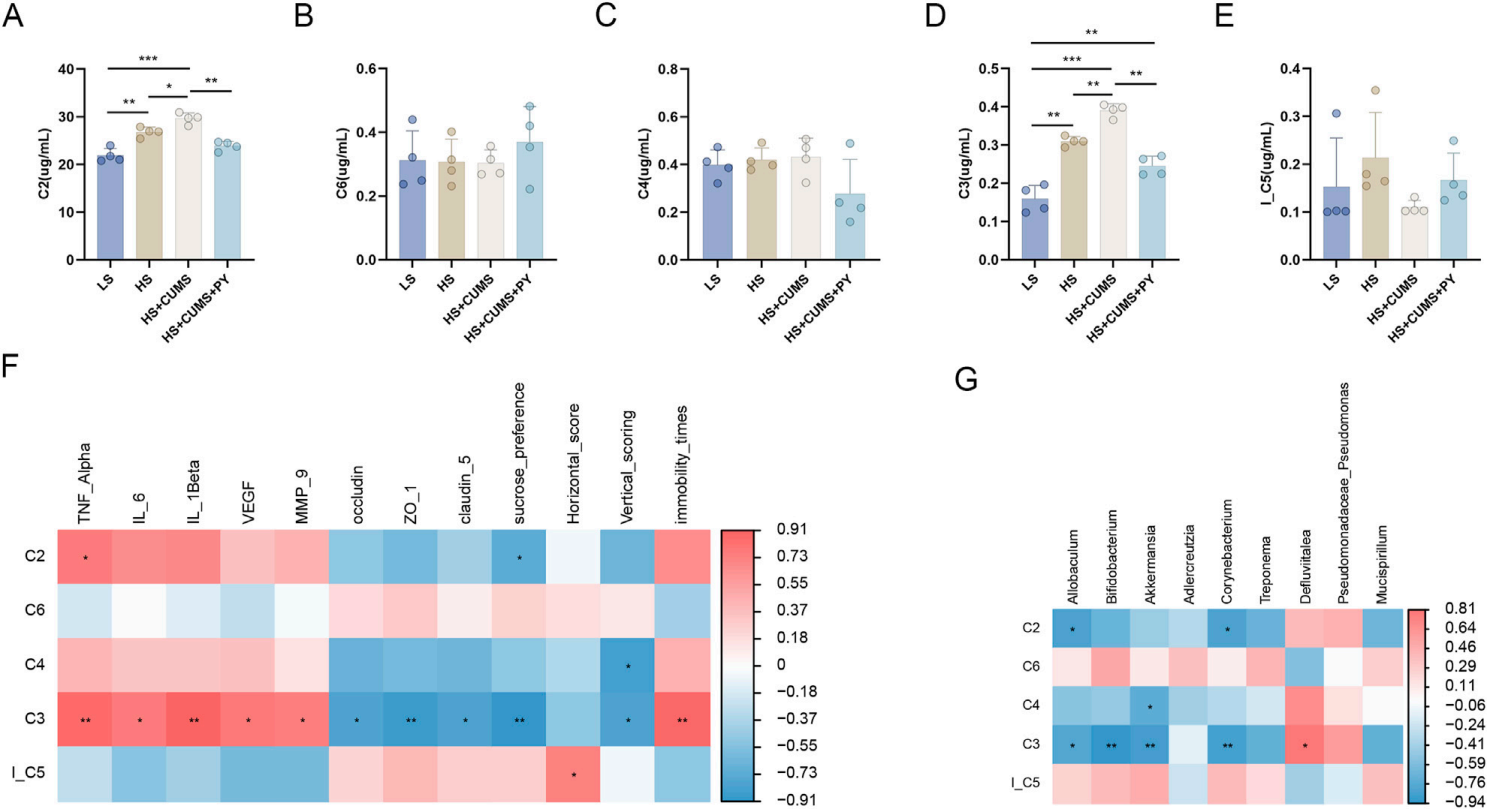
The relationship between metabolites and microbiota (*n* = 4). **(A–E)** Bar charts showing the changes in the levels of short-chain fatty acids acetic acid (C2), propionic acid (C3), butyric acid (C4), lsobutyric acid (I_C5), and caproic acid (C6) across different experimental groups (LS, HS, HS + CUMS, HS + CUMS + PY). **(F)** Heatmap depicting the correlation between short-chain fatty acids and environmental factors, including TNF-α, IL-β. **(G)** Heatmap presenting the association between short-chain fatty acids and signature microbiota. *P < 0.05, **P < 0.01, and ***P < 0.001.

Subsequently, we screened and identified the differential metabolites between the HS + CUMS group and HS + CUMS + PY group using orthogonal partial least squares (OPLS-DA), including acetic acid (C2), propionic acid (C3), butyric acid (C4), lsobutyric acid (I_C5), and caproic acid (C6) (Figures 7A-E). These metabolites (C2, C3, C4, I_C5, and C6) are short-chain fatty acids analyzed by gas chromatography-mass spectrometry (GC-MS). This analytical method allows for the precise identification and quantification of these important gut-derived metabolites. Spearman correlation analysis was then conducted to determine the relationships between these metabolites and environmental factors. We discovered that C3 was positively correlated with TNF-α, IL-6, IL-1β, VEGF, MMP-9, and immobility time yet negatively correlated with occludin, ZO-1, claudin-5 sucrose preference, and vertical scoring.

Furthermore, we also explored the connection between the metabolite and the signature microbiota. In the HS + CUMS + PY group, C3 was significantly associated with numerous microbes, such as *Allobaculum*, *Bifidobacterium*, *Akkermansia*, and *Corynebacterium* (Figures 7F, G). These results suggested that PY can regulate enrichment of the microbiota, thereby influencing the production of metabolites. This in turn can further modulate the inflammatory response and endothelial function in CSVD accompanied by depression.

### 2.5 Validation of the underlying mechanism of therapeutic effects of PY

We validated the microbiota-mediated mechanism of action of PY in CSVD accompanied by depression. Antibiotics were utilized to induce germ-free conditions in rats. Subsequently, the microbiota was transplanted from high-salt-fed rats subjected to CUMS. Recipient rats in the high-salt + CUMS + PY group were administered 1.407 g/kg/day of PY intragastrically for 4 weeks. We recorded the alterations in depressive-like behaviors, blood pressure, endothelial junction, and nerve integrity.

**FIGURE 8:**
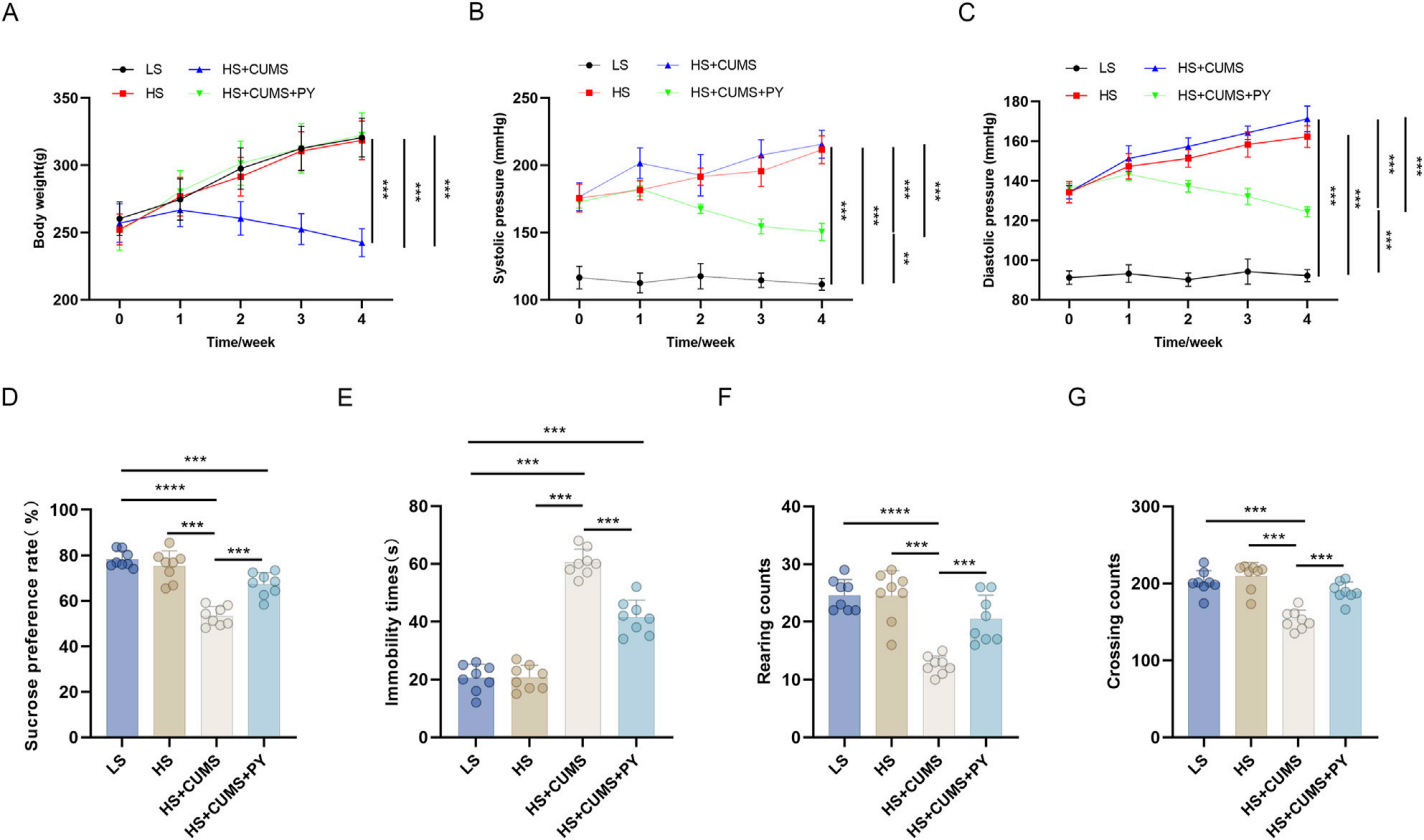
PY improved depressive-like behaviors in the fecal microbiota transplantation model (*n* = 8). **(A)** Body weight; **(B)** Systolic pressure; **(C)** Diastolic pressure; **(D)** Sugar preference; **(E)** Immobility time of the forced-swim test **(F)** Rearing count of the open-field test; **(G)** Crossing count of the open-field test. **P < 0.01, ***P < 0.001, and ****P < 0.0001.

We compared the recipient rats in the HS group and those in the HS + CUMS group. The rats in the HS + CUMS group exhibited depressive-like behaviors, manifested as a decrease in body weight (Figure 8A), sugar preference (Figure 8D), crossing frequency and rearing frequency (Figures 8F,G), as well as an increase in immobility time in FST (Figure 8E); Nevertheless, the systolic and diastolic blood pressure of the recipient rats in the two groups was comparable (Figures 8B,C). PY significantly reversed these changes in depressive-like behaviors, and regulated the blood pressure of the rats in the HS + CUMS group to a normal level (Figure 8).

**FIGURE 9:**
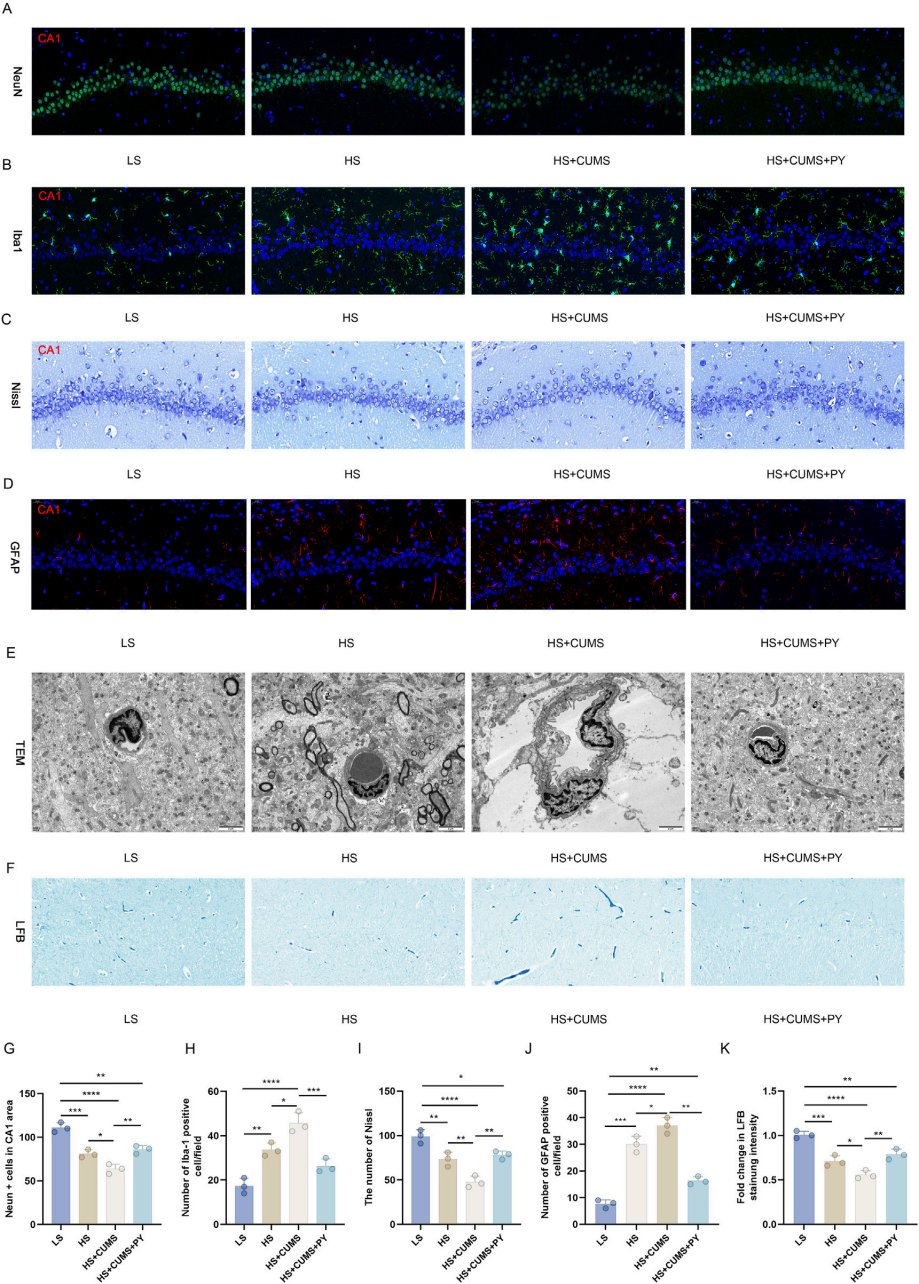
PY mitigates neuronal damage in the fecal microbiota transplantation model. The microbiota-mediated mechanism of action of PY in CSVD accompanied by depression was validated using pseudo germ-free rats. The body weight and depressive-like behaviors of the rats were measured. **(A)** Neurons labeled with NeuN; **(B)** Microglia labeled with IBA-1; **(C)** Nissl staining for the detection of Nissl corpuscle; **(D)** Astrocytes labeled by GFAP; **(E)** Transmission electron microscopy for the assessment of vascular endothelium; **(F)** LFB staining for myelin sheath; **(G)** NeuN positive cells in the CA1 region; **(H)** The number of IBA-1 positive cells; **(I)** Number of Nissl bodies; **(J)** Number of GFAP positive cells; **(K)** Fold change in the intensity of LFB staining. For quantitive data presented in **(G–K)** the *n* = 3 was applied. *P < 0.05, **P < 0.01, ***P < 0.001, and ****P < 0.0001.

Fluorescence images of different experimental groups were obtained to assess various cellular or molecular changes. As shown in Figure 9, in the LS group, a stable and relatively high expression of NeuN was detected, indicating a normal neuronal integrity. Besides, IBA-1 expression remained at a basal level, Nissl bodies presented a typical distribution pattern, and GFAP expression was moderate. All these indicators were within the normal range, suggesting that the cellular structure of the nervous system is intact, metabolism is normal, and the immune response is stable.

The microbiota derived from in the high-salt + CUMS donors reduced the number of NeuN-labeled neurons (Figure 9A) and increased the number of microglia labeled by IBA-1 (Figure 9B). This indicates that neuronal death and microglial activation occurred as a result of microbiota transplantation. Moreover, this microbiota caused a decrease in the number of Nissl corpuscles (Figure 9C) and the destruction of integrity of vascular endothelium (Figure 9D). The expression of GFAP in the HS + CUMS group was significantly elevated, indicating a strong activation of astrocytes (Figure 9D). Moreover, the impact of the microbiota also led to a decrease in white matter, with decreased myelin sheath (Figure 9E). Treatment with PY reversed the detrimental effects of microbiota from donors on a high-salt diet and exposed to CUMS. PY rescued the neurons (Figure 9A) and promoted the inactivation of microglia (Figure 9B). Additionally, PY alleviated destruction of vascular endothelium, increased the number of Nissl corpuscles and myelin sheath (Figures 9C–E). Furthermore, PY regulated the expression of GFAP, bringing it closer to the normal level and suggesting a restoration of astrocyte function (Figure 9D).

**FIGURE 10:**
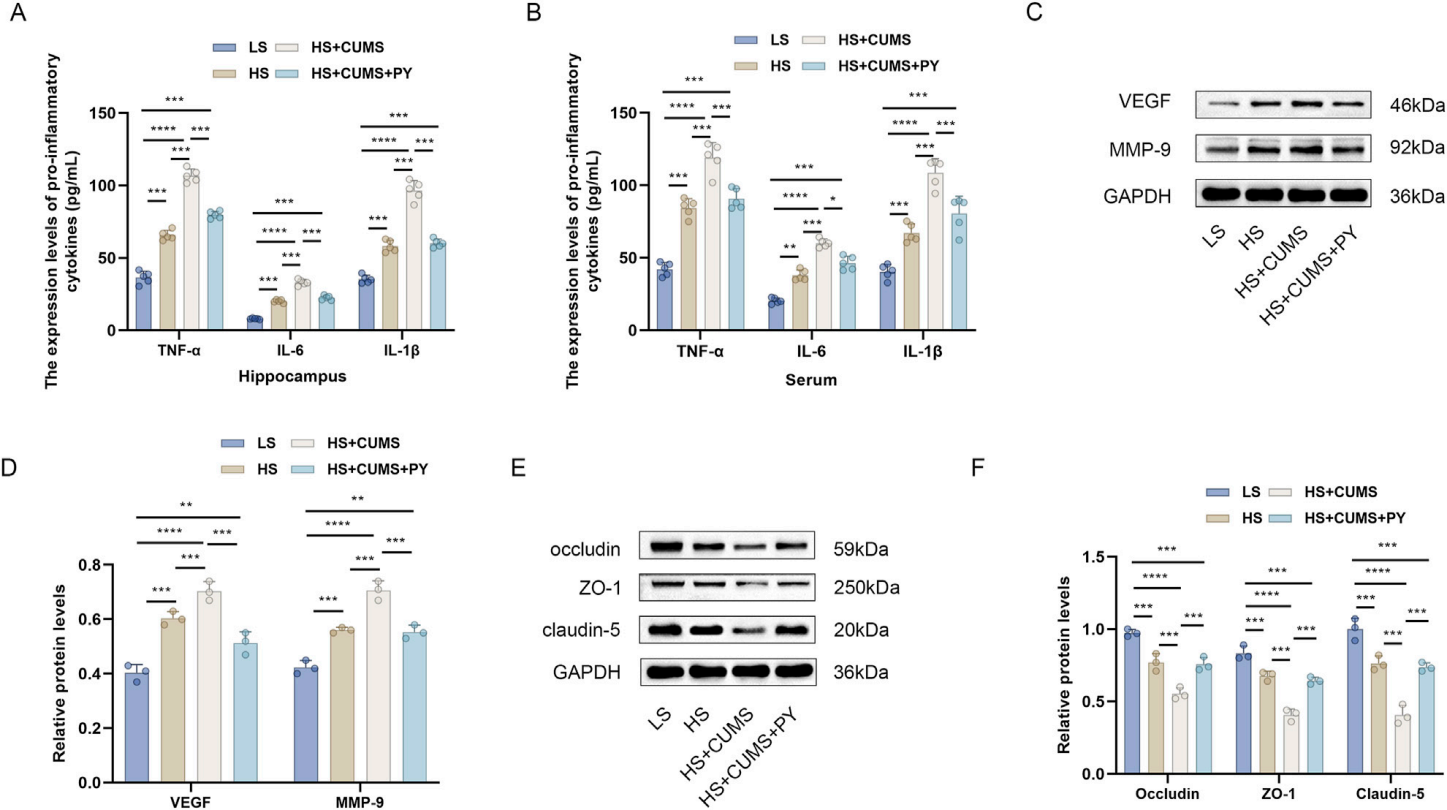
PY ameliorated endothelial dysfunction in the fecal microbiota transplantation model. **(A)** The expression levels of pro-inflammatory cytokines in the hippocampus were accurately determined using ELISA (*n* = 5); **(B)** The levels of pro-inflammatory cytokines in the serum were also determined by ELISA (*n* = 5); **(C)** Western blotting assays were employed to determine the expression level of VEGF and MMP-9 in the hippocampus; **(D)** The protein levels of VEGF and MMP-9 were presented (*n* = 3); **(E)** Western blotting assays were utilized to determine the expression level of occludin, ZO-1, and claudin-5 in the hippocampus; **(F)** The expression of occludin, ZO-1, and claudin-5 protein levels (*n* = 3). **P < 0.01, ***P < 0.001, and ****P < 0.0001.

The microbiota originating from the donors in the high-salt group induced substantial inflammation. Consequently, there was a notable elevation in the levels of TNF-α, IL-1β, and IL-6 within the hippocampus (Figures 10A,B). Moreover, the microbiota from high-salt donors undergoing CUMS further exacerbated the inflammation state (Figures 10A,B).

In contrast, treatment with PY led to a significant reduction in the levels of TNF-α, IL-1β, and IL-6 both in the hippocampus and serum (Figures 10A,B). Additionally, PY effectively reversed the dysregulation of VEGF, MMP-9, occludin, ZO-1, and claudin-5 in the recipient rats that had received the microbiota transplantation from high-salt-fed donors with CUMS. Specifically, PY decreased the levels of VEGF and MMP-9 while increasing the levels of occludin, ZO-1, and claudin-5 (Figures 10D-F). These findings strongly suggest that PY exerts a protective effect on the endothelial junction.

**FIGURE 11:**
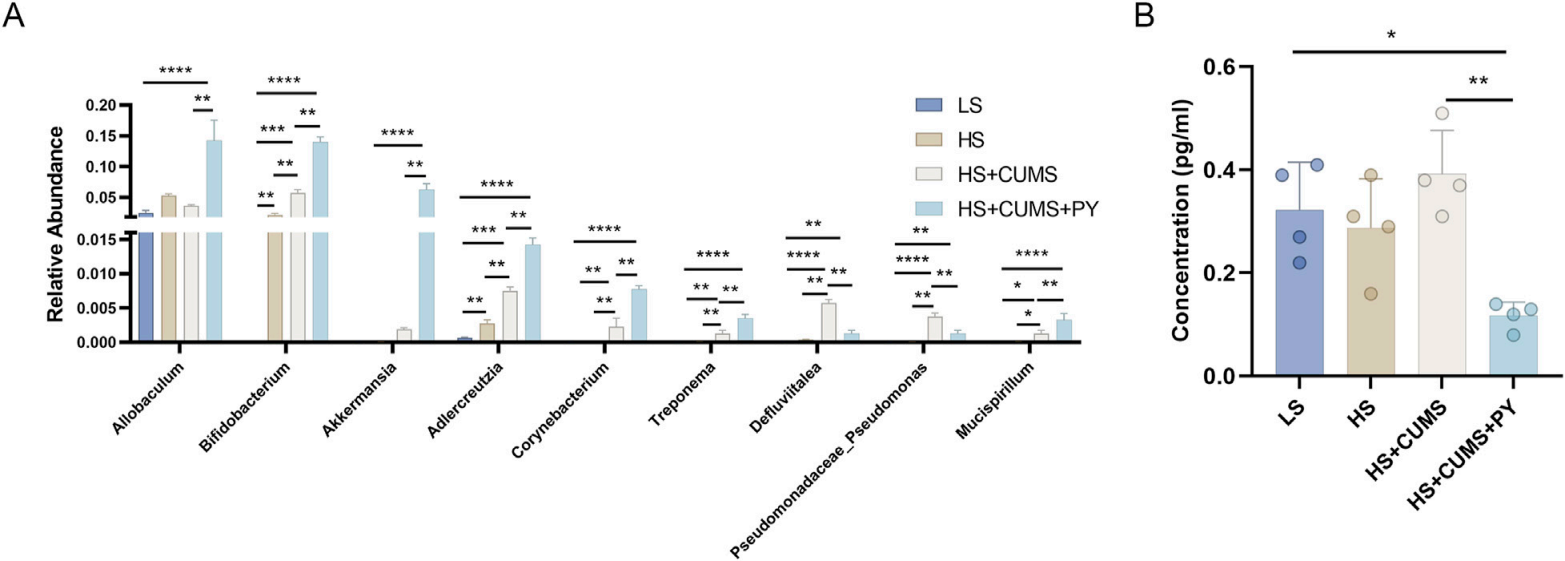
PY altered the signature microbiota and metabolites (*n* = 4). **(A)** The relative abundance of signature microbiota; **(B)** The level of signature microbiota in the serum. **P < 0.01, ***P < 0.001, and ****P < 0.0001.

The quantification analysis of the abundance of signature microbiota in recipient mice was carried out. Notably, these microbes were remarkably enriched in the group treated with HS + CUMS + PY (Figure 5). As a result, in the recipient mice of the HS + CUMS + PY group, the abundances of *Allobaculum*, *Bifidobacterium*, and *Akkermansia* were significantly higher compared to other groups. Additionally, the abundances of *Adlerceutzia*, *Corynebacterium*, *Treponema*, and *Mucispirillum* were also elevated in the HS + CUMS + PY group, even though the difference in abundance did not reach statistical significance (Figure 11A). Moreover, PY significantly reduced the concentration of C3 in these recipient mice (Figure 11B).

## 3 Discussion

Depression is a prevalent complication of CSVD. Effectively intervening in and treating the depressive symptoms associated with CSVD remains a formidable challenge. Several studies have recognized the pivotal role of the microbiota-gut-brain exerts its functions through complex pathways (Shi et al., 2023). These involve the vagus nerve within the nervous system, the influence of the neuroendocrine axis, and the activation of inflammatory and immune cells in the gut, which are triggered by various factors (Shi et al., 2023). Consequently, we investigated the antidepressant effects of PY in CSVD and evaluated its mechanism of action on the crosstalk among endothelial function, microglial cells, inflammation, and gut microbiota. A rodent CSVD model was established by inducing hypertension via a high-salt diet, followed by the induction of depressive symptoms using CUMS. We discovered that PY ameliorated depressive-like behaviors, alleviated endothelial dysfunction, and suppressed microglial activation and inflammation in CSVD rats with comorbid depression. PY might mediate these protective effects by regulating the gut microbiota.

Initially, PY was found to inhibit depressive-like behaviors in the rat model of CSVD accompanied with depression. This was determined by an increase in the body weight, an increase in the number of crossings and rearing counts in the OFT, an increase in sugar preference and a decrease in the immobility time in the FST. Some medicinal components in PY exhibited antidepressant effects. For example, ginsenoside Rg1, an extract of *Panax ginseng*, has the ability to reverse the depressive symptoms in rats suffering from depression induced by chronic restraint stress (CRS) (Li et al., 2022b). The underlying mechanism is associated with the GAS5/EZH2 mediated activation of microglial cells and the occurrence of mitochondrial dysfunction. *Panax notoginseng* and its main active components can regulate neurodevelopment, neurotransmitters, and neuroinflammation to suppress depressive symptoms (Xie et al., 2018). These medicinal components of PY can exert anti-depressant effects in the nervous system.

Endothelial dysfunction is the primary contributing factor in the development of CSVD (Bai et al., 2022). The vascular endothelium plays a pivotal role in vascular homeostasis by regulating vasoactive molecules, such as pro-inflammatory cytokines (Wang et al., 2022). Impaired endothelial function can increase the permeability of the BBB and compromise its function, thereby enabling these cytokines to infiltrate the brain (Low et al., 2019). Microglial activation occurs when pro-inflammatory mediators enter the brain (Gao and Hernandes, 2021), which may further exacerbate damage to the BBB, intensify inflammation and contribute to depression (Ronaldson and Davis, 2020; Fang et al., 2023). Therefore, we evaluated the effects of PY on microglial activation, inflammation, and endothelial function in the rat models of CSVD with comorbid depression. Our results indicated that PY alleviated nerve damage and suppressed microglial activation. Additionally, PY also decreased the levels of pro-inflammatory cytokines (VEGF and MMP-9) and increased the levels of tight junction-related proteins, suggesting that PY mitigated endothelial dysfunction. In summary, PY may alleviate depressive symptoms in CSVD rats by alleviating vascular endothelial injury.

High-salt treatment may enhance the susceptibility to depression. Our results demonstrated that although high-salt treatment did not induce depressive-like behaviors in rats, it impaired the integrity of the nervous system and the cerebral endothelial barrier. These alternations may contribute to the absence and dysfunction of the complex emotional circuits (Lima-Ojeda et al., 2018; Fox and Lobo, 2019). Thus, although hypertension induced by high-salt treatment did not directly cause depression, it provided a histopathological environment for the occurrence and development of depression. Although PY reversed the impairment of neurons and endothelial cells, thereby inhibiting depression, it failed to improve hypertension in rats. We hypothesized that the improvement in nerves and vessels induced by PY might not be sufficient to counteract the changes induced by hypertension in these aspects.

The gut microbiota was found to mediate the anti-depressive effects of PY in CSVD. Alternations in the gut microbiota are a significant contributing factor to depression (Eltokhi and Sommer, 2022). Previous studies have shown that animals with CUMS-induced depression exhibit a high abundance of certain bacteria, such as *Prevotellaceae* and *Desulfovibrio*, which are detrimental to health (Wu et al., 2021). A high abundance of *Bacteroides thetaiotaomicron*, *Klebsiella oxytoca*, and *Klebsiella aerogenes* also contributes to the occurrence and development of antibiotic-induced depression (Fan et al., 2022). Zhao et al. found that the gut microbiota can modulate the activation of the BDNF/CREB pathway in the hippocampus to induce depression (Zhao et al., 2022). We discovered that rats treated with PY had a distinct microbiota composition compared to those suffering from CSVD with depression. Altered gut microbiota provide the target for drug treatment. Based on LEfSe, *Allobaculum*, *Bifidobacterium*, *Akkermansia*, *Adlerceutzia*, *Corynebacterium*, *Treponema*, and *Mucispirillum* were identified as biomarkers of PY treatment, and *Pseudomonas* and *Defluviitalea* were found to be the signatures of CSVD with depression. Thus, the microbiota can be regarded as an anti-depressive target of PY in the progression of CSVD. We investigated the correlation between these biomarkers and 12 environmental factors. *Allobaculum* and *Akkermansia* showed stronger correlations with the environmental factors influencing CSVD with depression.

The relationship between *Allobaculum* and the tight junction proteins has been reported previously. *Allobaculum* can upregulate the expression of tight junction proteins by producing butyrate (Zhou et al., 2022). In addition, a positive correlation has been identified between the endothelial tight junctions and *Allobaculum*. By means of correlation analysis, it was determined that *Allobaculum* exhibits an anti-inflammatory effect on CSVD accompanied by depression. Butyrate-producing bacteria are known to play a positive and significant role in the treatment of depression (Valles-Colomer et al., 2019; Ngo and Vo, 2019). Our research findings revealed that *Allobaculum* is positively associated with the improvement of depressive-like behaviors in rats. Specifically, the enrichment of *Allobaculum*, which is induced by PY, can restore the tight junction structure in the endothelial barrier. This restoration not only inhibits the intrusion of foreign substances but also has an ameliorative effect on depressive-like behaviors. *Allobaculum* is an important probiotic of the gut microbiota, playing a key role in regulating metabolism and inflammation (Cani et al., 2022). During our research, we further discovered an inverse correlation between *Allobaculum* and the level of inflammation. Notably, the abundance of bacterium *Allobaculum* was significantly higher in rats treated with PY. Moreover, a positive correlation was observed between the increased abundance of *Allobaculum* and alleviation of depressive behaviors. In summary, these results strongly suggested that bacterium *Allobaculum serves* as the target of PY at the microbial level. Therefore, PY has the potential to recruit beneficial microbiota in the gut, enabling the gut microbiota for the treatment of diseases related to inflammation and depressive symptoms.

We discovered that PY could inhibit the blood pressure increase caused by high-salt diet and long-term stress, indicating a protective effect of PY on rats afflicted with CSVD accompanied by depression. This phenomenon likely stemmed from alterations in gut microbiota. These diseases may possess distinct microbiota phenotypes. It is probable that PY induced the enrichment of microbiota related to depression rather than that related to hypertension in rats. As a result, PY might have solely improved depressive-like behaviors by safeguarding the vessels and nerves, without exerting any influence on hypertension. Further investigations are required to elucidate the relationship between diseases and gut microbiota.

We also measured the levels of metabolites in the blood and determined the metabolites that were significantly associated with depressive symptoms. In the experimental model of CSVD complicated by depression, we observed that the treatment with PY led to a reduction in the levels of C3. C3, which shows a positive correlation with TNF-α, IL-6, and IL-1β, can suppress inflammation in the context of CSVD with depression. Additionally, C3 plays a crucial role in cerebral sphere dysfunction, and it is positively correlated with VEGF and MMP-9, but negatively correlated with occludin, ZO-1, and claudin-5. Besides, it was negatively correlated with sucrose preference and vertical scoring but positively correlated with immobility time. Therefore, C3 can promote depressive-like behaviors in the experimental model of CSVD with depression. C3 may be a contributing factor to depression during CSVD and a potential target for PY treatment. In this study, PY reduced C3 levels, thereby inhibiting neuroinflammation and cerebral area dysfunction, and ultimately improving the depressive-like behaviors of rats. Gut microbiota can produce metabolites that are transported to the brain via the bloodstream. Microbiota-mediated metabolites can modulate the pathogenesis of depression. Alterations in the microbiota, such as amino acid disorders caused by changes in the gut microbiota, can interfere with neurotransmitters in the prefrontal cortex, resulting in depressive-like symptoms (Xie et al., 2022). For the first time, we confirmed the relationship between C3 and gut microbiota. C3 was negatively associated with biomarkers of PY treatment, including *Allobaculum*, *Bifidobacterium*, *Akkermansia*, and *Corynebacterium*, but positively associated with *Defluviitalea* (a biomarker of CSVD with depression). The correlation between gut microbiota and C3 indicates that PY can alter the signature microbiota to drive the production of metabolites that modulate C3-meditated neuronal inflammation, endothelial dysfunction, and depressive-like behaviors.

In summary, PY has the potential to ameliorate depressive symptoms, alleviate endothelial dysfunction, and inhibit microglial activation and inflammation, thereby impeding the progression of CSVD complicated by depression. Notably, over-activated microglia and inflammation are strongly associated. The beneficial effects of PY are likely attributed to its modulation of metabolite production by the gut microbiota. Our findings suggest that PY could serve as a novel therapeutic agent for the treatment of CSVD with depression, which may facilitate the development of more effective treatment strategies in the future.

## 4 Methods

### 4.1 Animals

Dahl/Salt-sensitive rats were purchased from Beijing Vital River Laboratory Animal Technology Co., Ltd. (Beijing, China). Before the experiments, the rats were allowed *ad libitum* access to water and a diet containing 0.3% NaCl. They were housed in a controlled environment maintained at a temperature of (21 ± 1)°C and a relative humidity of (59 ± 1)%, with a 12 h light/dark cycle.

### 4.2 Establishment of rat models of CUMS complicated by depression and treatment regiments

After a 1-week acclimation period, rats were randomly divided into five groups (*n* = 8 rats per group). The groups were designated as follows: the low-salt (LS) group, high-salt (HS) group, high-salt + CUMS (HS + CUMS) group, high-salt + CUMS + PY (HS + CUMS + PY) group, and high-salt + CUMS + escitalopram (HS + CUMS + ESC) groups. The purpose was to mimic cerebral microvascular disease.

Determination of dosage of PY: The dosage of the traditional Chinese medicine PY, administered to rats was determined based on the clinical dosage in humans. Clinically, the PY granule comes in a sachet of 6.7 *g*, and the recommended daily intake for humans is two sachets. Considering an average adult body weight of 60 kg, the human dosage per kg of body weight is calculated as (6.7 g *2)/60 kg. According to the widely-recognized conversion factor of 6.3 for dose extrapolation from humans to rats in pharmacological research, the dosage of PY for rats was determined as [(6.7 g *2)/60] *6.3 = 1.407 g/kg.

For 4 weeks, the rats were provided with a diet containing 8% NaCl. The rats in the LS group were fed a diet with 0.3% NaCl, which served as the control for the high-salt group. To induce cerebral microvascular disease accompanied by depression, the rats on the high-salt diet were simultaneously exposed to chronic unpredictable mild stress (CUMS) following a previously described protocol (Li H. et al., 2021). Stress stimulations included swimming at 4°C for 5 min, water deprivation for 24 h, tail-suspension for 5 min, cage tilting at 45° for 24 h, restraint for 30 min, exposure to a moist environment for 24 h, and shaking for 10 min. The rats were subjected to one of these stress stimuli daily for 4 weeks, with the daily stimulation randomly selected during CUMS period. For drug treatment, during the CUMS modeling process, the rats were administered 1.407 g/kg per day of PY or 1.05 mg/kg per day of ESC via intragastric administration for 4 weeks. Here, ESC (H. Lundbeck A/S, Denmark) was used as the positive control for PY.

### 4.3 Fecal microbiota transplantation

Prior to fecal microbiota transplantation (FMT), the recipient rats were administered a cocktail of antibiotics, including 1 g/L ampicillin, 1 g/L metronidazole, 0.5 g/L vancomycin, and 0.5 g/L neomycin for 5 days. To verify the clearance of flora in the internal environment of pseudo-germ-free rats, feces from the control group and the experimental group were collected, and were inoculated onto suitable culture media for 3 days. Once the pseudo-germ-free rat model was successfully constructed, as confirmed by the above - mentioned methods, the rats can be utilized for further experiments.

In accordance with a method detailed in another study (Wu et al., 2020), FMT was carried out to verify the microbiota-mediated role of PY in CSVD with depression. Briefly, fecal samples were respectively collected from donor rats in the LS, HS, and HS + CUMS groups, respectively. The recipient rats were divided into four groups (8 rats/group): LS, HS, and HS + CUMS, and LS, HS, and HS + CUMS + PY groups.

For FMT, 100 mg of fecal samples were homogenized in 1 mL of sterile saline, followed by centrifugation at 800 *g* for 3 min. Then, the obtained supernatant was used as the transplant material. To maintain the stability of the bacterial composition, fresh transplant material was prepared on the day of transplantation and administered orally 10 min after preparation. The transplantation was performed weekly for four consecutive weeks using an oral gavage. After FMT, rats in the high-salt + CUMS + PY group were treated with 1.407 g/kg/day of PY, while other groups were treated with distilled water via oral administration for 4 weeks.

### 4.4 Weight and blood pressure measurement

Body weight was measured weekly prior to the blood pressure test. Diastolic and systolic blood pressures were measured using a blood pressure monitor on a weekly basis as well (Panlab, United States).

### 4.5 Depressive-like behavior test

#### 4.5.1 Sugar preference test

To acclimatize the rats to the experimental environment, each rat was initially placed in a clean cage furnished with two bottles filled with 1% sucrose solution for a day. On the subsequent day, these two bottles were replaced with two bottles of pure water. During the implementation of the sucrose preference test, a bottle containing 1% sucrose solution and another bottle containing pure water were placed in the cage. Each rat was permitted unrestricted access to the two bottles for a day. The alternations in the weights of the two bottles were recorded (W1: change in the weight of the 1% sucrose solution bottle; W2: change in the weight of the pure water bottle). The sucrose preference = [W1/(W1+W2)] × 100%. Prior to the experiment, the rats were deprived of food and water for 24 h. A decrease in sugar preference indicated the presence of depressive-like states in the rats.

#### 4.5.2 forced-swim test

Each rat was compelled to swim in a glass cylinder (diameter: 20 cm; height: 50 cm) filled with water (35 cm high water column) at (24 ± 1)°C for 15 min for the initial acclimation process. After a 24-h acclimation period, the rats were once again subjected to forced swimming in the same glass cylinder for 6 min. During this 6-min period, the time with the first 2 min was designated as the mobility time, while the time in the subsequent 4 min was recorded as the immobility time in the forced-swim test (FST). An elevation in the immobility time served as an indication that the rats manifested depressive-like behaviors.

#### 4.5.3 Open-field test

During the acclimation phase, each rat was placed into a plastic chamber (72 × 72 × 40 cm) equipped with a ceiling, and let undisturbed in a silent environment for 30 min. This initial acclimation period was crucial to ensure the rats’ comfort within the experimental setup. After a full 24-h acclimation, each rat was positioned at the exact center of the chamber. Subsequently, the rats were allowed to move about freely for a period of 4 min. During this time, the number of squares traversed by one of the rat’s limbs was recorded as the crossing count. Simultaneously, the duration for which the rat’s hind limbs were in a standing position was accurately noted and registered as the rearing count. These recorded parameters would serve as essential behavioral indices for further analysis.

### 4.6 Immunofluorescence

Upon completion of the last behavioral test, the rats were humanely euthanized via cervical dislocation. Subsequently, the cornu ammonis 1 (CA1) regions of their hippocampal tissues were precisely dissected, and were fixed and embedded in paraformaldehyde for subsequent processing.

To study the cellular composition and distribution in the CA1 region of the hippocampus comprehensively, three key markers were utilized: NeuN for neurons, IBA-1 for microglia, and GFAP for astrocytes.

First, under the microscope, the hippocampal slices were carefully examined to identify the CA1 region. The embedded tissues containing the CA1 area were then sectioned into 3-mm thick slices. These slices underwent a series of procedures. They were dewaxed and hydrated using a standard protocol with xylene and ethanol solutions of decreasing concentrations. After that, they were incubated in 1 mM EDTA solution (pH = 8.0) (P0085, Beyotime, Shanghai, China) at 95°C for 12 min. Once cooled to room temperature, the sections were fixed with 4% paraformaldehyde (P0099, Beyotime, Shanghai, China) at room temperature for 15 min, and then treated with blocking buffer (P0102, Beyotime, Shanghai, United States) for 1 h. Diluted primary antibodies, including anti-NeuN antibody (ab177487, 1:3000, Abcam, United States), anti-IBA-1 antibody (ab178846, 1:2000, Abcam, United States), and anti-GFAP antibody (ab134436, 1:200, Abcam, United States) were added to the sections. The slides were then incubated overnight at 4°C in the dark. On the following day, anti-rabbit IgG H&L (ab150077, 1:500, Abcam, United States) antibodies were incubated with the sections at room temperature for 1 h. Finally, the labeled neurons by NeuN and microglia by IBA-1 were observed under a fluorescence microscope (Olympus, Japan).

### 4.7 Luxol fast blue (LFB) staining

After undergoing dewaxing and hydration procedures, the hippocampal tissue sections (3 mm thick) were incubated with a pre-heated LFP staining solution at 60°C for 2 h. Once the incubation was complete, the sections were allowed to cool down to room temperature. Subsequently, they were incubated with 95% ethanol for 2 min, distilled water for 30 s, 0.05% LiCO_3_, 70% ethanol, and distilled water for another 30 s, respectively. Following dehydration, the sections were mounted in neutral balsam. Finally, the morphology of the myelin sheath was observed under a microscope (Olympus, Japan).

We used Image-Pro Plus software (Media Cybernetics, Inc. United States) to conduct a further analysis of the staining results. The specific measurement indices include staining intensity, which is quantified by reading the pixel grayscale values, and the area of the positive-staining region, automatically identified and calculated by this software.

### 4.8 Nissl staining

After dewaxing and hydration, the hippocampal tissues sections (3 mm thick) were carefully placed in Nissl staining solution (Beyotime, Shanghai, United States). Incubation occurred at a precisely maintained temperature of 37°C for a duration of 10 min. The sections were incubated with 95% ethanol twice and then with xylene twice. The sections were embedded in neutral balsam for observation under the microscope.

### 4.9 ELISA

ELISA was performed to determine the levels of pro-inflammatory cytokines, including TNF-α, IL-1β, and IL-6. The rats were anesthetized with 3% isoflurane. Subsequently, 0.4 mL of blood was collected from the abdominal aorta of rats and placed in the tube without the anticoagulant. The blood was kept at 37°C until coagulation occurred. Then it was centrifuged at 3,000 rpm for 10 min. The supernatant was collected, and ELISA was performed using a kit (TNF-α: ab236712, Abcam, United States; IL-1β: ab255730, Abcam, United States; IL-6: R6000B, R&D system, United States). In addition, 1 *g* of hippocampal tissue was homogenized in 9 mL of PBS containing a proteinase inhibitor. After that, the homogenate was centrifuged at 5,000 *g* for 10 min at 4°C. The levels of TNF-α, IL-1β, and IL-6 in the supernatant were measured using specific ELISA kits for TNF-α (RAB0480-1KT, Sigma-Aldrich, United States), IL-1β (RAB0278-1KT, Sigma-Aldrich, United States), and IL-6 (RAB0312-1KT, Sigma-Aldrich, United States), respectively.

### 4.10 Western blotting analysis

The hippocampal tissue lysate in RIPA buffer (P0013B, Beyotime, Shanghai, China) was centrifuged at 10,000 *g* for 5 min to determine the loading amount. For the electrophoresis procedure, the BCA protein assay kit (P0012S, Beyotime, Shanghai, United States) was employed to measure the protein concentration in the supernatant. The total proteins were separated by SDS-PAGE (Bio-Rad, United States), and then, the protein bands in gels were transferred onto PVDF membranes (Millipore, United States). The membranes were incubated with the blocking buffer (P0023B, Beyotime, Shanghai, United States) for 1 h, followed by an overnight incubation with diluted primary antibodies at 4°C. The primary antibodies used included anti-VEGF, anti-MMP-9, anti-occludin (ab216327, 1:1000, Abcam, United States), anti-ZO-1 (40-2200, 1:1000, Invitrogen, United States), anti-claudin-5 (PA5-94924, 1:5000, Invitrogen, United States), and anti-GAPDH (ab181602, 1:10000, Abcam, United States). GAPDH was used as the internal reference for the target proteins. The secondary antibodies (ab205718, 1:10000, Abcam, United States) were used to culture the membranes for 1 h at 37°C. Subsequently, the membranes were reacted with an ECL kit (P0018S, Beyotime, Shanghai, China) for 1–2 min. An X-ray imaging system (Bio-Rad, United States) was used to obtain the blot images, and the gray values were required using the ImageJ software.

### 4.11 Transmission electron microscopy

The hippocampal tissues were incubated in 2.5% glutaraldehyde for 2 h, and then, in 1% osmium tetroxide for 1 h. Subsequently, these samples were dehydrated at room temperature using 30%, 50%, 70%, 80%, and 90% acetone solutions respectively, with each dehydration step lasting for 30 min. The tissue samples were then embedded in epoxy resin for 36 h at 60°C. The tissue sections (50 nm thick) were stained with a lead citrate solution. The subcellular structures were observed using a transmission electron microscope (JEOL, Japan).

### 4.12 16S rDNA sequencing

Fresh fecal samples (2 *g*) from rats were placed in an aseptic tube and frozen in liquid nitrogen for 15 min. Then, these samples were stored at −80°C. The DNA in the fecal samples was extracted using the PowerFecal^®^ DNA Isolation Kit (Qiagen, GER). The 16S rDNA sequencing was performed by Personalbio Technology Co., Ltd. (Shanghai, China). Sequencing was performed using the Illumina platform with paired-end sequencing. The V3-V4 region of 16S rDNA was amplified using a forward primer (5′-ACT CCT ACG GGA GGC AGC A-3′) and a reverse primer (5′-GGA CTA CHV GGG TWT CTA AT-3′).

### 4.13 Microbiome bioinformatics analysis

Microbiome bioinformatics analysis was performed using the QIIME2 tool. The raw paired-end sequence data were first demultiplexed using the demuxplugin, followed by primer trimming with the cut adapt plugin. Then, the sequences were merged, filtered, and dereplicated using DADA2. Genes related to species taxonomy were annotated according to the Greengenes database. Chimera sequences in the amplicon sequences were removed. The Chao1 (for species richness), Simpson (for species diversity), and Shannon (for species diversity) indices were calculated to characterize the α-diversity of gut microbiota. The β-diversity was evaluated by principal coordinate analysis (PCoA) based on weighted UniFrac distance. A *T*-test was performed to identify the different microbiota communities between groups. Linear discriminant analysis coupled with effect size measurements (LefSe) was used to identify signature microbiota. Based on the KEGG database, the phylogenetic investigation of communities by reconstruction of unobserved states (PICRUSt) tool was used to explore the functions of the altered gut microbiota. Hierarchical clustering analysis was performed according to the unweighted pair-group method with arithmetic means.

### 4.14 Gas chromatography-mass spectrometry

After the rats were anesthetized with 2% isoflurane, blood samples were collected from the abdominal aorta of the rats and placed into heparin-containing tubes. Subsequently, these tubes were centrifuged at 3,000 rpm for 10 min at 4°C. Next, 0.2 mL of supernatant was pipetted into 2 mL tubes and then frozen in liquid nitrogen for 10 min; the frozen tubes were stored at −80°C until further analysis. The short-chain fatty acids in the samples were determined by GC-MS using an Agilent 7890B-5977A system (Agilent, United States). The samples were separated on a DB-5MS column (length: 30 m; internal diameter: 0.25 mm; film thickness: 0.25 μm) (Agilent, United States) with helium flow rate at 1 mL/min. During the GC-MS analysis, the ion source temperature was set at 150°C and the cold trap temperature was set at 230°C. The temperature program for the gas chromatography was as follows: initially, the temperature was held at 60°C for 30 min. Then, it was increased at rate of 8°C/min to 125°C, followed by an increase at a rate of 5°C/min to 210°C, then at a rate of 10°C/min to 270°C, and finally, at a rate of 20°C/min to 305°C. The final temperature of 305°C was maintained for 5 min. Mass spectrometric data were acquired in scan mode over a mass-to-charge ratio (m/z) range of 50–500.

### 4.15 Orthogonal partial least squares-discriminant analysis (OPLS-DA)

Different metabolites were determined by setting the threshold of *p* < 0.05 and variable importance of projection (VIP) > 1. For the metabolomics analysis, metabolites were regarded as upregulated when its fold change was >1, and downregulated when the fold change was <1. OPLS-DA was carried out using the R muma package.

### 4.16 Statistical analysis

The data obtained from animal experiments were presented as the mean ± SEM and analyzed using SPSS 22. GraphPad Prism software was employed to visualize the data prior to conducting the comparison analysis. First, the Shapiro-Wilk test was applied to the data to determine whether it followed a normal distribution. Then, the differences in the data among the groups were evaluated by performing a one-way analysis of variance (ANVA), followed by Dunnett’s multiple comparison test. All differences among and between groups were classified according to the *P*-value levels: *P* < 0.05 was considered statistically significant; *P* < 0.01 indicated a highly significant difference; *P* < 0.001 represented an extremely significant difference; and *P* < 0.0001 signified a very extremely significant difference.

## Data Availability

The original contributions presented in the study are publicly available. This data can be found here: 10.6084/m9.figshare.29264477.

## Supplementary material

SUPPLEMENTARY FIGURE S1 Verification of flora clearance in pseudo-germ-free rats (*n* = 3). **(A)** Petri dish images of samples from the control group and pseudo-germ-free rats. **(B)** Bar chart representing the colony forming units (CFU) of the control group and pseudo-germ free rats. The “**” indicates a significant difference (*P* < 0.01).

SUPPLEMENTARY FIGURE S2 Effects of PY on the physiological and behavioral parameters of rats in different experimental groups (*n* = 8). **(A)** The body weight trends in different treated groups; **(B)** The systolic changes in different treated groups; **(C)** The diastolic changes in different treated groups; **(D)** Source performance rate in different treated groups. **(E)** Crossing counts in different treated groups; **(F)** Rearing counts in different groups. **P* < 0.05, ***P* < 0.01, ****P* < 0.001, and *****P* < 0.0001.
