# Supplementary figures and images for: The anti-depressive role of the Pei Yuan Kai Yu formula in cerebral small vessel disease based on gut microbiota
Source: Front Pharmacol. 2025 Jun 13;16:1510250. doi: 10.3389/fphar.2025.1510250 (PMC12202353; doi:10.3389/fphar.2025.1510250)

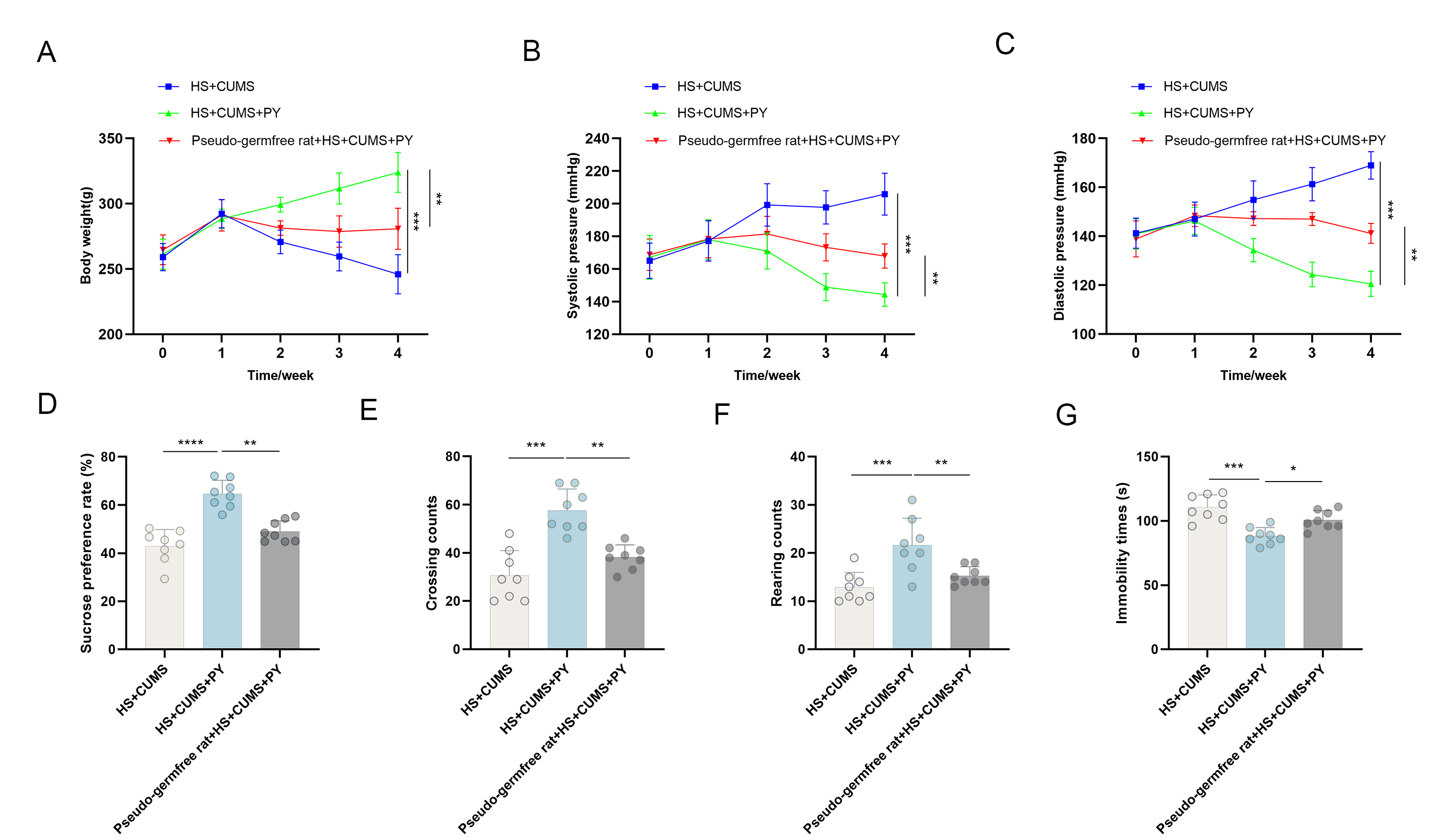

Supplement: Supplementary file 1 [file Image2.tif]

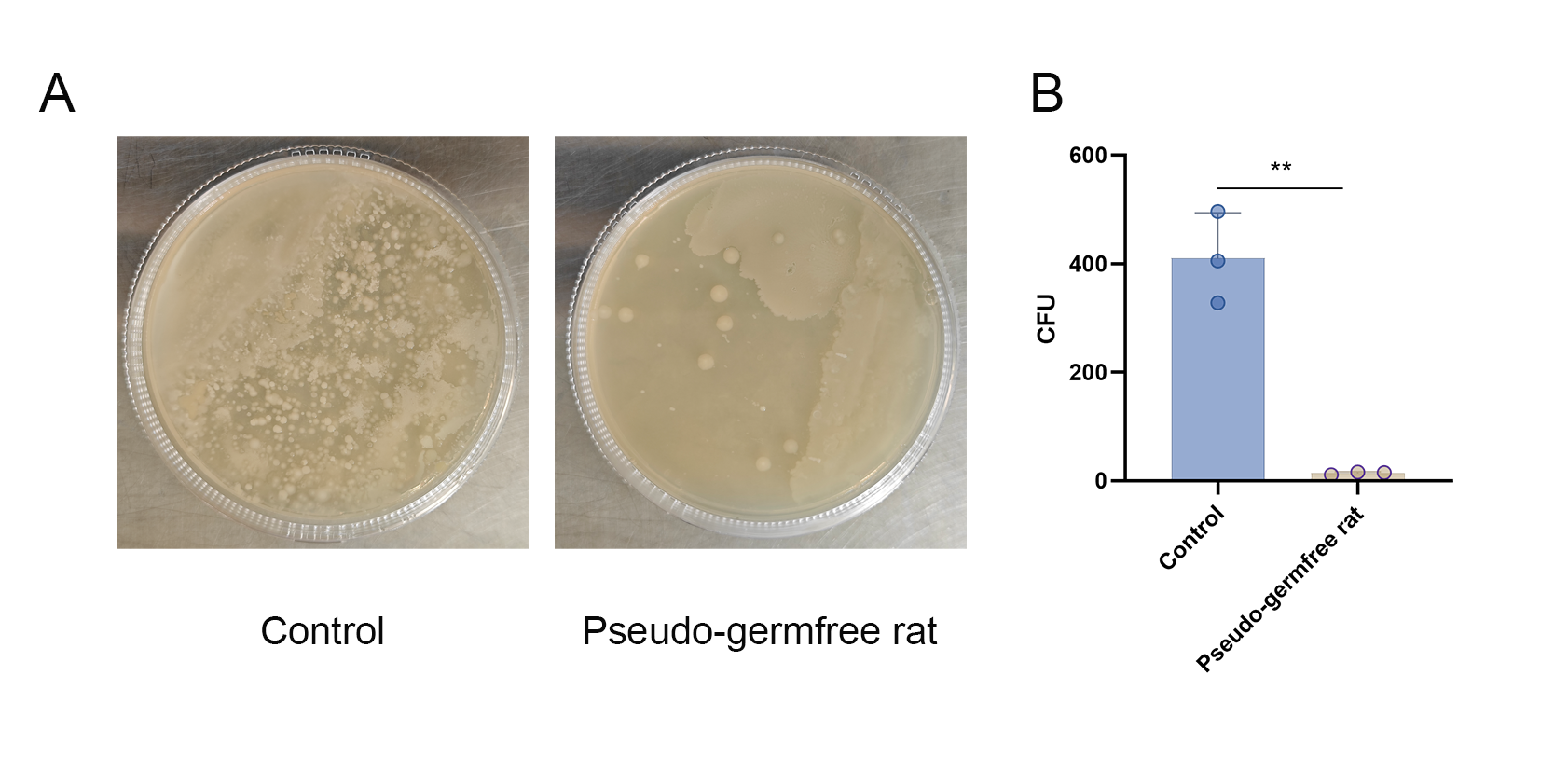

Supplement: Supplementary file 2 [file Image1.tif]
